# Global phylogenetic structure of the hyperdiverse ant genus *Pheidole* reveals the repeated evolution of macroecological patterns

**DOI:** 10.1098/rspb.2014.1416

**Published:** 2015-01-07

**Authors:** Evan P. Economo, Pavel Klimov, Eli M. Sarnat, Benoit Guénard, Michael D. Weiser, Beatrice Lecroq, L. Lacey Knowles

**Affiliations:** 1 Okinawa Institute of Science and Technology Graduate University, 1919–1 Tancha, Onna-son 904–0495, Japan; 2 Department of Ecology and Evolutionary Biology, Museum of Zoology, University of Michigan, 830 N University Street, Ann Arbor, MI 48701, USA; 3 Tyumen State University, 10 Semakova St., Tyumen 625003, Russian Federation; 4 Department of Entomology, University of Illinois at Urbana–Champaign, 320 Morrill Hall, 505 S. Goodwin Avenue, Urbana, IL 61801, USA; 5 Department of Biology, University of Oklahoma, 730 Van Vleet Oval, Norman, OK 73019, USA

**Keywords:** diversification, formicidae, species richness, radiation, body size, phylogenetic niche conservatism

## Abstract

Adaptive radiations are of particular interest owing to what they reveal about the ecological and evolutionary regulation of biodiversity. This applies to localized island radiations such as Darwin's finches, and also to rapid radiations occurring on a global scale. Here we analyse the macroevolution and macroecology of *Pheidole,* a famously hyperdiverse and ecologically dominant ant genus. We generate and analyse four novel datasets: (i) a robust global phylogeny including 285 *Pheidole* species, (ii) a global database on regional *Pheidole* richness in 365 political areas summarizing over 97 000 individual records from more than 6500 studies, (iii) a global database of *Pheidole* richness from 3796 local communities and (iv) a database of *Pheidole* body sizes across species. Analysis of the potential climate drivers of richness revealed that the patterns are statistically very similar across different biogeographic regions, with both regional and local richness associated with the same coefficients of temperature and precipitation. This similarity occurs even though phylogenetic analysis shows that *Pheidole* reached dominance in communities through serial localized radiations into different biomes within different continents and islands. *Pheidole* body size distributions have likewise converged across geographical regions. We propose these cases of convergence indicate that the global radiation of *Pheidole* is structured by deterministic factors regulating diversification and diversity.

## 1. Introduction

Documenting and understanding global biodiversity patterns has long been central tasks of basic and applied ecological research. In recent years, there has been an unprecedented aggregation and synthesis of biodiversity data providing a highly resolved ‘biodiversity map’ for some well-studied taxonomic groups [1–5]. In addition, many recent phylogenetic studies have provided evolutionary context for extant biodiversity patterns, as well as new insights into the processes underlying those patterns [6–11]. However, there remain large gaps in our knowledge. Global, comprehensive biodiversity datasets are available only for plants and certain vertebrate groups [1–5], excluding the majority of species on the Earth—invertebrate animals. There is much to learn about whether invertebrate faunas inhabiting similar biomes but geographically distant regions originate from phylogenetic niche conservation [12] and long-distance dispersal, or whether lineages evolved *in situ* within different regions to adapt to different habitats. Moreover, the main factors responsible for large-scale climate–richness correlations continue to be hotly debated and explored empirically [13,14]. In particular, the extent to which macroecological patterns are determined by fundamental constraints on ecological and evolutionary processes (e.g. geographical variation in speciation rates, richness–energy relationships), or are mostly due to contingent historical factors (e.g. glaciation history, climate cycles), remains unclear [15,16].

Here, we address these issues with an integrated analysis of macroevolutionary and macroecological structure of the hyperdiverse ant genus *Pheidole*. Among invertebrates, ants are of particular interest as they are dominant components of most terrestrial ecosystems, both in terms of biomass and in the functional roles they play in community (e.g. seed dispersal) and ecosystem (e.g. nutrient cycling) processes [17]. Recent work has resolved the deeper structure of the ant phylogeny [18,19] and led to insights about the broad patterns of ant diversification [20], but much work remains to resolve and understand diversification patterns within individual genera. Among the 323 ant genera, *Pheidole* is one of the two most species-rich (along with *Camponotus*, which is probably not monophyletic), with approximately 8% (1124/14 747) of described ant species [21]. *Pheidole* species are found in most temperate and tropical biomes and every continent except Antarctica, and known to be particularly dominant in tropical habitats.

We investigate whether regional *Pheidole* faunas are assembled through dispersal, in which lineages conserve their climate affinities and colonize different biomes around the world, or whether *Pheidole* environmental niche evolves rapidly, filling niche space within each region. Simultaneously, we ask whether macroecological climate–richness and body size distributions vary across geographical regions. The joint analysis of whether regional faunas are evolved through dispersal or *in situ* radiation, and whether the distribution of species across niche space (climate or body size) evolves to be similar in each region, is informative on whether the evolution of such patterns are deterministic or contingent on historical factors.

Deterministic evolution leads to similar outcomes when beginning from similar starting points, while contingent evolution can lead to different outcomes as chance events influence the outcomes of evolutionary dynamics. The study of spatially replicated evolutionary radiations in a phylogenetic context has been particularly informative about the roles of chance and determinism in evolution [22–30]. For example, recent work has revealed stunning cases of convergent evolution of community structure across different islands [22,29], in which a particular assemblage of species' ecologies and morphologies evolves repeatedly. The presence or absence of such convergence indicates which features of biodiversity patterns and community structure may be more inevitable, for example, because of the structure of adaptive landscapes that underlie trait evolution [22].

We can similarly investigate large-scale macroecological patterns such as climate–richness correlations. Like the ecological and morphological structure of communities arranged across a set of islands, macroecological biodiversity patterns reflect the distribution of diversity across niche-space, and the nature of such patterns can reflect underlying organizing principles or historical effects [15,16,31]. Recent work has made great strides aggregating macro-scale biodiversity data and analysing environmental and historical factors correlated with biodiversity patterns [1–5]. However, there remains a lack of integration between phylogenetic analyses of individual radiations and aggregate analyses of macroecological patterns. It is generally unclear, for example, whether macroecological patterns represent highly probable outcomes shaped by strong underlying ecological and/or evolutionary constraints, or whether they are driven by historical contingencies. Even when macroecological patterns can be compared across regions, it may be difficult to untangle the roles of phylogenetic niche conservatism and evolutionary determinism in explaining similarity across regions. In many species-rich groups, including all of those where global richness datasets are available, large-scale macroecological patterns are not evolutionarily independent owing to interchange of lineages across geographical regions. Thus, even when macroecological patterns are similar across regions, it could be due to deterministic constraints on their formation, or simply owing to a shared (and possibly contingent) evolutionary history.

Despite the global distribution of *Pheidole*, previous phylogenetic work suggested that there is a high degree phylogenetic separation between Old and New World faunas [19,32], with most Old World taxa descended from a single colonist from the New World. However, there was fairly sparse sampling of Old World species included in that analysis, and the only temperate Old World species (*Pheidole pallidula*) was recovered as an independent colonization of the New World, leaving open the question of whether different biomes were colonized through intercontinental dispersal or evolutionary niche shifts in each region. To address this question, we reconstructed a new global phylogeny of 285 *Pheidole* species in a multilocus species-tree framework, including sampling from all continents and biogeographic realms, extending previous work on *Pheidole* phylogenetics [19,32]. If regional *Pheidole* faunas have indeed evolved through localized radiations that fill environmental niche space, it provides an opportunity to evaluate whether similar macroecological patterns re-evolve in different regions.

To quantify the macroecological structure of *Pheidole* diversity, we compiled an exhaustive accounting of known *Pheidole* species distributions across 365 political regions by aggregating literature records and specimen databases. We also assembled a complementary database on *Pheidole* richness from over 3500 local communities across the globe. Using these datasets, we analysed species richness–climate relationships, a pattern that reflects the underlying distribution of species across climatic niche space. To our knowledge, this represents the first global analysis of species richness patterns in an invertebrate group based on comprehensive literature records. In addition to climate–richness patterns, we analysed macroecological variation in one of the most fundamental phenotypic traits: body size.

With these combined analyses, we sought to establish whether (i) macroecological climate–richness and body size distributions are similar in different regions across the globe, and (ii) whether any similarity in the way biodiversity is distributed across niche space is due to phylogenetic niche conservatism and dispersal assembly of regional faunas or re-evolution of the patterns during within-realm *in situ* radiation and filling of climate niche-space. More generally, we ask whether the global *Pheidole* dominance, diversity and niche distribution are characterized by determinism leading to repeatable outcomes, or whether they are consistent with a more contingent view of evolution and ecology, where biogeographic patterns are shaped by historical factors with different outcomes in different regions.

## 2. Material and methods

### (a) Taxon sampling

We assembled a broad geographical base of material through our own collections and generous donations, and performed additional sequencing using DNA extractions from two previous molecular phylogenetic studies on *Pheidole* [19,32]. There are a total of 513 sequenced ant individuals (294 species) in our data matrix; 314 were our specimens, 74 were borrowed DNA extractions through the courtesy of C.S. Moreau (Field Museum of Natural History, Chicago; [19,32]), and 125 were GenBank sequences from the same studies that were vetted for possible errors (electronic supplementary material, table S1). Our new material included large collections from New Guinea, Australia, New Caledonia, Micronesia/Palau, the Solomon Islands, Southeast Asia and representatives of Fischer's species groups in Africa [33]. Our new samples were concentrated in the Old World *Pheidole*, but as we used several new markers since previous phylogenetic work, we also sequenced additional loci from the DNA extracted from previous molecular studies [19,32] from both Old and New World taxa.

For new DNA extractions, our taxon selection process involved several rounds of identification and sequencing. From an initial pool of 800 collections, we point-mounted two major–minor sets (if available), totalling over 2300 specimens. We then sorted material samples to species/morphospecies and sequenced cytochrome oxidase 1 (CO1) from at least two samples of each putative morphospecies (if available), totalling 513 individuals. Using the CO1 data to assist further morphological analysis, we refined our identifications and morphospecies definitions, and reduced our initial set to 200 samples for which we sequenced two additional loci and combined the data from previous studies. From this set, we sequenced six additional loci (nine in total) from 65 representative taxa selected from all major lineages identified with the larger taxon dataset. We used this combined dataset to select two taxon sets for analysis; the 64 taxa with nine genes sequenced, and a second more comprehensive 285-species dataset including one sample per species, with the criterion that at least three loci were available for each sample. Some additional species with fewer than three loci were included in the 285-taxon set if they were consistently placed in species groups near close relatives in the preliminary analyses and if that placement is consistent with morphology (see electronic supplementary material, table S2 for details about loci sequenced for each specimen included in phylogenetic analyses).

Identifications were based on published literature, comparison with specimens (including type specimens) from collections at the Museum of Comparative Zoology (MA, USA), California Academy of Sciences (CA, USA), Los Angeles County Museum (CA, USA), Natural History Museum of Geneva (Geneva, Switzerland), Hungarian Natural History Museum (Budapest, Hungary) and private collections. Voucher specimen images were taken of each species or morphospecies included in our study (excluding taxa from previous DNA extractions for which we had no specimens). All data (more than 2500 records) and images (more than 1000) for specimens used in this project are available on Antweb (www.antweb.org).

### (b) DNA extraction, amplification and sequencing

Genomic DNA was isolated from a single specimen or its legs using a QIAamp DNA Micro kit (Qiagen) with the manufacturer's protocol, except that specimens were pierced with a fine needle (but not crushed) to preserve the specimen, and the incubation time (proteinase K lysis) was extended to 24 h.

Nine loci (*CO1*, *His3.3B* (Histone H3.3B F1 copy), *Lop1* (long wavelength sensitive opsin 1), *GRIK2* (glutamate receptor ionotropic, kainate 2-like), *unc_4* (unc-4 homeodomain gene), *LOC15* (uncharacterized locus *LOC15*), *CAD1* (conserved ATPase domain protein gene), *EF-1*α* F2* (elongation factor 1-alpha F2) and *Top1* (DNA topoisomerase 1); total of 8820 bp (aligned)) were amplified using nested PCR, which is tolerant to very low amounts of template DNA and poor sample quality. Six loci (CO1, His3.3B, Lop1, CAD1, EF-1α F2, Top1) were selected from published studies [18,19,32,34,35], two loci (GRIK2, unc_4) were selected based on the lowest sequence similarities between genomic sequences of *Atta cephalotes* [36] and *Acromyrmex echinatior* [37], and one anonymous locus (LOC15) was selected based on our unpublished next-generation sequencing data (E. P. Economo, P. Klimov, E. M. Sarnat, B. Guénard, M. D. Weisers, B. Lecroq and L. L. Knowles 2012, unpublished data). For all nine loci, oligonucleotide primers were designed in Primer3 [38] using five ant genomes from GenBank to amplify long fragments using less degenerate primers than those for amplifying targeted segments of loci to avoid parologous loci. Because ant genomes contain two copies of the genes *EF1-α* and *His3.3B*, protocols were designed to amplify copy-specific templates for these genes: F2 for *EF1-α* and F1 (designated here) for *His3.3B*. Amplification protocols and primer sequences are given in electronic supplementary material, table S2. In order to prevent the amplification of numts (non-functional mitochondrial pseudogenes in the nuclear genome), we used a long PCR spanning adjacent mitochondrial loci to recover the targeted CO1 locus. Amplicons were sequenced in both directions by the University of Michigan DNA Sequencing Core. All sequences were deposited in GenBank (Accession Nos. KJ141206–142138; electronic supplementary material, table S1). Alignments of most loci were unambiguous (i.e. no gaps); a few regions were excluded to avoid erroneous homology assignment owing to gaps in the sequences. The data matrix containing the alignment is available from TreeBASE (http://www.treebase.org, submission ID: 16494).

### (c) Phylogenetic reconstruction

We took a Bayesian approach to all phylogenetic reconstruction. For the nine-locus representative 65-taxon dataset, we conducted a species-tree analysis with the program *BEAST [39]. Models of nucleotide substitution were selected based on AICc in PartitionFinder v. 1.1.1 [40]: GTR + Γ (unc_4, LOC15), HKY + Γ + I (CAD1) and GTR + Γ + I (all others). For each locus, substitution, clock and tree models were unlinked. We used the default strict clock for every partition. Ploidy was set to 0.5 for the mitochondrial CO1 gene tree and 2.0 for the eight nuclear gene trees. The species tree prior was set to Yule process, the population size model prior was assigned to piecewise linear and constant root; and the clock rate prior was set to uniform for every partition. We ran 10 separate runs with the same start file on the TOMBO cluster at the Okinawa Institute of Science and Technology, each for around 4.6 × 10^10^ generations sampling every 10 000 states. Based on examination of the trace files, the burnin was set conservatively at 10 million states, yielding approximately 3600 post-burnin trees per run sampled from the posterior. Effective sample size (ESS) values for likelihoods and all other parameters exceeded 200 for the combined runs. The trees were combined and thinned by half in the program LogCombiner, resulting in a sample of approximately 17 000 trees from the posterior. We used TreeAnnotator to find the maximum clade credibility tree across this set, which was analysed as our result. To check for consistency of results, MCC trees from different individual runs were compared and were found to be nearly identical with some minor variation in node posterior probability values.

Several *Pheidole* fossils are known from Baltic and Dominican ambers, but none can currently be assigned to, or excluded from, clades within the phylogeny of extant *Pheidole*, thus there are no calibration points other than a minimum age on the root node of our phylogeny. Thus, to date our species tree inferred with *BEAST, we used a recently published date of 59.8 Ma for the most recent common ancestor of *Pheidole* and the Cephalotini inferred as part of a wider study of the Myrmicinae subfamily [41]. The latter study incorporates information from molecular divergence and fossils from all related genera, and gives a more robust age than could be inferred in our taxonomically more limited analysis.

For the larger analysis of 285 species, a number too large for *BEAST, we used the MPI version of MrBayes v. 3.2.1 [42] to analyse the concatenated data, with nucleotide substitution parameters partitioned by gene and assuming a GTR + Γ + I model of nucleotide substitution. We ran two runs with four chains each on the TOMBO cluster until the standard deviation of split frequencies fell below 0.01 using a relative burnin of 0.25 generations. Stopping criteria were met after 6.25 × 10^10^ generations, and a 50% majority rule consensus tree was computed. We ran several instances of the analysis to confirm consistency of results, but the analysed result presented in this paper was taken from a single run.

### (d) Macroecological data compilation and analysis

The macroecological data used in this paper were assembled as part of the Global Ant Biodiversity Informatics (GABI) project led by several of the authors. The GABI database contains literature data from over 6500 published studies, and specimen databases including ANIC/CSIRO and Antweb [43], with the lowest geographical resolution provided for each record. The database, constituting over 1 million records (more than 97 000 for *Pheidole*), summarizes known distribution for all ant species at the global scale, and will be described in detail in a subsequent paper.

First, we used these data to compile a *Pheidole* species list and richness count for each of 365 political regions, following the scheme for political regions used in a previous paper [44]. We only considered records for identified, described *Pheidole* species and excluded records known to be due to human introductions (e.g. the global spread of *Pheidole megacephala*) from the richness count. Climate variables were estimated by taking the spatial mean for each region for mean annual temperature (MAT) and mean annual precipitation (MAP) using BioClim data at 10-min resolution [45].

*Pheidole* is a taxonomically challenging genus with many undescribed species and uncertainties on the species level. As such, distributional data based on species names will inevitably be incomplete and imperfect. Although broad-scale climate–richness patterns should be relatively robust to taxonomic uncertainty, we sought to mediate this potential effect by including an independent measure of taxonomic effort as a potential covariate of richness in each political region (see next section). Second, we also performed an analysis of richness patterns using data from local communities, which does not depend on correct species identification (only an accurate local morphospecies count).

To compile the community dataset, we used the same set of 6500 references and identified 1060 studies that reported community composition and *Pheidole* richness for one or more local communities, resulting in a dataset of richness from 3793 local communities distributed across the globe. Other variables relative to the location (latitude and longitude), habitat types, collection methods used or the type of strata sampled (leaf-litter, ground or arboreal) were also noted.

We applied the following filters to the community data: we excluded studies that (i) did not employ methods that targeted ground-foraging ants, (ii) were from habitats that are impacted by humans, (iii) detected fewer than 10 total species or (iv) used only active sampling methods such as baiting, which left 1473 of the initial 3792 communities. As some studies involved sampling many communities in a localized area, and as we are examining broad patterns at large scales, we further rarefied the data to both minimize microvariation in richness and pseudoreplication. We grouped communities with a single-linkage clustering algorithm [46], resulting in 705 clusters. In this method, all pairs of sites within a distance threshold (in this case, 10 km) are considered ‘linked’, and clusters are contiguous networks of sites (i.e. each pair is connected by a direct linkage or indirect path through intermediate sites). We then only used the community with the highest *Pheidole* richness in each cluster for the analysis, which left 705 data points for the analysis.

For each of the geographical localities sampled, we obtained climatic data from the WORDCLIM database using the 2.5 arc-min resolution dataset [45], and extracted variables related to temperature and precipitation regime using DIVA-GIS. Biome type was assigned by cross-referencing sampling localities for sequenced specimens with the Terrestrial Ecoregions of the World dataset [47] using Arc-GIS.

#### (i) Statistical analyses

We used Poisson regression in a Generalized Linear Models (GLM) framework to model macroecological variation in *Pheidole* richness across climatic gradients. We focused on the effects of two climate variables that best predict the distribution of biomes across the planet (as encapsulated in the classic Whittaker plot [48]) and are often highly correlated with species richness of many taxa including ants [49–51]: mMAT and MAP. As variation in richness of described species across political regions is likely to be influenced by the area of each region as well as sampling and taxonomic effort, we used area and an index of undersampling as additional variables in the analysis. As our index of undersampling, we used the number of ‘missing’ genera in the published literature for each political region, as estimated by a previous study [44]. Because climate–richness relationships are often asymmetric between the Northern and Southern Hemispheres for a broad range of taxa [52], including ants [49], we also fit hemispheric models for richness. The community data were analysed similarly using Poisson regression in a GLM framework, but without area (because they are point measurements) and sampling deficit (as there were no independent data to estimate deficit) as additional variables.

### (e) Body size data

We assembled a dataset of *Pheidole* images for species included in the phylogeny using the online database Antweb (www.antweb.org), Wilson's monograph [21] and a set of approximately 1000 new extended depth of field photographs taken at the Okinawa Institute of Science & Technology with a DFC400 camera mounted on a LeicaM205C stereomicroscope. As part of a broader project on *Pheidole* morphological evolution, we developed systems of homologous two-dimensional landmarks for ants imaged in the three standard views used on Antweb: head (12 landmarks), profile (six landmarks) and dorsal (four landmarks). We aligned landmarks for each view using a Generalized Procrustes Analysis using the program TpsDig [53], and used the log-transformed scale factor as our index of body size. In lieu of a true body mass measurement, which is difficult for small organisms such as ants, the landmark scaling factor is more robust than using a single length measurement, as it incorporates the geometric relationships of several homologous points. We only used the average of the scaling factor for head and profile views because a calibrated image of the dorsal view was missing for some species. All data were taken from minor workers to maximize coverage over species in our molecular dataset, but as body size of minors is highly correlated with body size of majors (Spearman's *ρ* = 0.91, *n* = 213, *
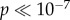
*), this represents the main axis of body-size variation across species.

## 3. Results

### (a) Global phylogenetic structure of *Pheidole*

**Figure 1.**
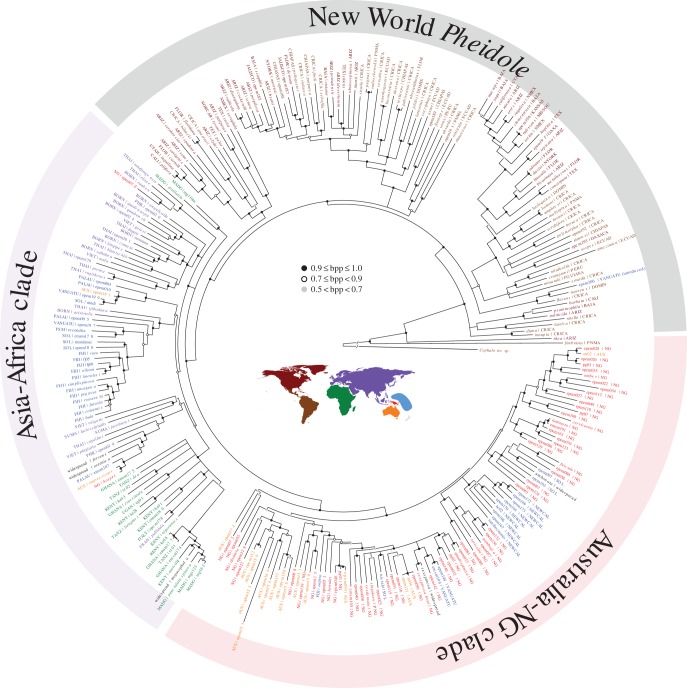
Global phylogenetic structure of *Pheidole*, annotated by geography. The tree is a 50% majority rule consensus topology from a posterior sample of trees generated by a Bayesian analysis with MrBayes v. 3.2.1, and node symbol reflects Bayesian posterior probability (bpp).

Our phylogenetic analyses 285 taxon set with concatenated data (gene-tree estimated with MrBayes for 285 taxa; figure 1) and nine-locus dataset with exemplar sampling of clades (species-tree estimated with *BEAST for 66 taxa; electronic supplementary material, figure S1) yielded resolved, broadly consistent phylogenies. With the stem age of *Pheidole* fixed at 59.8 Ma, the crown age of *Pheidole* is estimated to be 37.3 Ma (electronic supplementary material, figure S1). The estimated phylogenetic relationships were in agreement with previous studies that recovered *Pheidole fimbriata* and *Pheidole rhea* as basal to the rest of *Pheidole* [19]. Both the species-tree and concatenated analysis recovered the monophyly of all Old World *Pheidole* with the crown node dated to 22.1 Ma, nested within New World *Pheidole*. The single exception is a species related to *Pheidole moerens* and *Pheidole flavens* collected from Vanuatu, which was almost certainly introduced recently by humans (see [54]). A previous study had inferred that the European species *P. pallidula* represented an additional colonization of the Old World [19], rather than being nested within the African *Pheidole.* Our re-analyses of the data found that this was due to inclusion of paralogous copies of the gene *His3.3B* in at least several taxa that led to erroneous inferences.

In the Old World, the *Pheidole* phylogeny shows a high degree of geographical structure with one clade comprised of African and Asian groups (henceforth, AF–ASIA clade), and a second comprised primarily of Australian and New Guinean groups (AUS–NG clade). One lineage represented by two Malagasy species, *Pheidole grallatrix* and *Pheidole* mg110, is recovered as either sister to the AUS–NG clade in the species tree, or in a three-way polytomy with the AUS–NG clade and AF–ASIA clade in the concatenated analysis. Whether the majority of the diverse Malagasy *Pheidole* species are related to the *P. grallatrix* lineage, or belong to other African (or non-African) species groups, remains for future work.

The *Pheidole* faunas on the archipelagoes of Melanesia and Micronesia are assembled through a mixture of colonizing lineages from the Asia–Africa clade and the AUS–NG clade. The *Pheidole* communities of the most remote archipelagoes of Fiji and New Caledonia are notable in being primarily assembled through *in situ* speciation rather than through the accumulation of species through dispersal. Other than the presence of several widespread species distributed across the Pacific (*P. megacephala*, *P. oceanica, P. fervens, P. sexspinosa, P. umbonata*), the Fijian community was assembled through colonization of one lineage from the Asian–African clade and subsequent radiation into 10 species and colonization of another single lineage (*Pheidole onifera*) from the AUS–NG clade. All ten New Caledonian *Pheidole* species included in our analysis were descended from a single colonist from the AUS–NG clade.

### (b) Macroecological structure of *Pheidole* diversity

**Figure 2.**
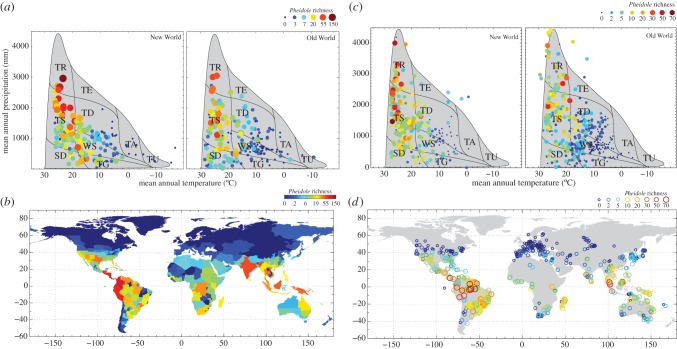
Variation in *Pheidole* species richness by political region (*a*,*b*) and in local communities (*c*,*d*) in climatic and geographical space. The locations of the Earth's biomes, based on Whittaker's classic graph [48], are superimposed on the plot of mean annual temperature and mean annual precipitation. The biomes depicted are tropical rainforest (TR), tropical seasonal forest/savanna (TS), temperate rainforest (TE), subtropical desert (SD), temperate deciduous forest (TD), woodland/shrubland (WS), taiga (TA), tundra (TU).

While *Pheidole* species can be found across a broad range of environmental conditions, there are strong correlations between species richness with climatic parameters. Across political regions, *Pheidole* richness shows positive correlations (table 1 and figure 2) with temperature (MAT), precipitation (MAP) and area, and negative correlations with sampling deficit (as represented by absence of generic records, see §2). The intercepts vary between the New World (NW) and Old World (OW), with NW regions having higher richness compared with regions with similar climates in the OW, even though the coefficients for NW and OW *Pheidole* richness with climatic variables are close in magnitude. When the model is fit to each hemisphere separately, there is a North–South asymmetric climate-richness relationship, with temperature having a relatively stronger influence than precipitation in the north than that in the south. In addition, the intercepts are generally larger in magnitude in the Southern Hemisphere for both OW and NW, indicating higher richness at a given climate in the south than the north. These North–South Hemispheric asymmetries are not uncommon across taxonomic groups [52], including ants [49].

**Table 1.** Fitted coefficients and standard errors from Poisson regression models for *Pheidole* richness across 365 political regions. The columns represent the parameters and s.e. of the parameters fitted to various subsets of the data.

coefficient	New World	Old World	NW Northern Hemisphere	OW Northern Hemisphere	NW Southern Hemisphere	OW Southern Hemisphere
intercept	2.14 ± 0.03	1.18 ± 0.05	2.09 ± 0.04	1.07 ± 0.05	2.37 ± 0.05	1.66 ± 0.13
MAP	0.32 ± 0.02	0.44 ± 0.02	0.31 ± 0.05	0.49 ± 0.03	0.27 ± 0.05	0.32 ± 0.04
MAT	0.89 ± 0.04	0.81 ± 0.05	1.10 ± 0.05	0.85 ± 0.05	0.47 ± 0.08	0.48 ± 0.15
expert deficit	−0.58 ± 0.03	−0.63 ± 0.05	−0.58 ± 0.04	−0.67 ± 0.06	−0.49 ± 0.06	−0.62 ± 0.09
area	0.25 ± 0.02	0.48 ± 0.03	0.40 ± 0.03	0.41 ± 0.04	0.25 ± 0.07	0.56 ± 0.09

Our analysis of the *Pheidole* richness in local communities (table 2 and figure 2) corroborates the overall pattern found in the political regions dataset. In both the Old World and New World, *Pheidole* richness is strongly correlated with MAT and MAP, and shows hemispheric asymmetry in the climate coefficients in the North–South, but not East–West, directions. While climate correlations are similar, *Pheidole* is generally a larger fraction of richness in the New World than Old World by a factor of about 1.5; in Neotropical wet forests, *Pheidole* constitutes 18% of local ant species richness, while that figure is 11% in Old World wet forests.

**Table 2.** Fitted coefficients and standard errors from Poisson regression models for *Pheidole* richness in local communities. The columns represent the parameters fitted to various subsets of the data.

coefficient	New World	Old World	NW Northern Hemisphere	OW Northern Hemisphere	NW Southern Hemisphere	OW Southern Hemisphere
intercept	1.74 ± 0.03	0.94 ± 0.03	1.64 ± 0.06	1.10 ± 0.04	1.75 ± 0.04	0.73 ± 0.06
MAP	0.15 ± 0.02	0.26 ± 0.02	0.19 ± 0.03	0.33 ± 0.03	0.23 ± 0.03	n.s.
MAT	0.71 ± 0.03	0.79 ± 0.04	0.46 ± 0.05	0.60 ± 0.05	0.88 ± 0.05	1.07 ± 0.15

### (c) Evolution in environmental space

**Figure 3.**
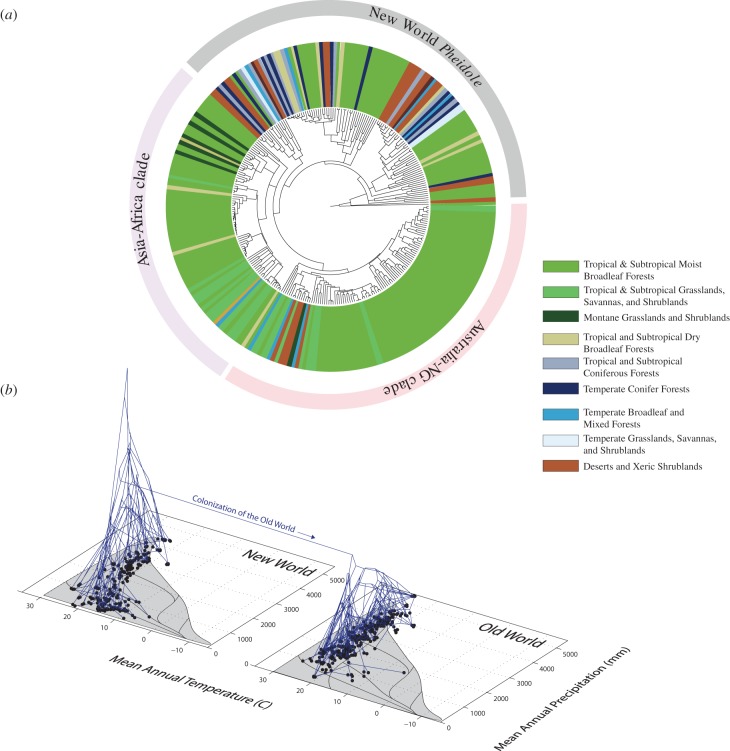
(*a*) The global phylogeny annotated by biome. Biomes are based on Olson *et al.* [47]. (*b*) Evolution of *Pheidole* in Whittaker's climate space. We plot locations of *Pheidole* specimens used in the phylogenetic analysis, and the phylogeny with reconstructed ancestral states on the vertical axis.

*Pheidole* species can be found in a range of environmental conditions and biomes. Our phylogenetic analysis shows that colonization of different habitat types occurred through evolutionary transitions within each biogeographic region rather than phylogenetic niche conservatism and dispersal between similar habitats across regions (figure 3). In other words, the global *Pheidole* radiation consists of a constellation of geographically localized radiations within the New World, Africa, Asia and Oceania, each of which involved evolution between a range of different biomes including warm wet tropical forests, deserts, savannahs and cooler temperate regions.

### (d) Body size evolution

Body size distributions are nearly identical for the New World and Old World *Pheidole* faunas, with the mean body sizes indistinguishable between the two areas (electronic supplementary material, figure S2). As the Old World *Pheidole* comprise a monophyletic clade, this implies a re-evolution of the distribution found in the New World. Moreover, the diversity in body size within both New World and Old World *Pheidole* faunas highlights the evolutionary lability of this trait. The similarity in size distributions does not merely reflect strong constraints on body size, but rather the independent evolution of a broad spectrum of body sizes in the New World and Old World (electronic supplementary material, figure S2).

## 4. Discussion

From their origins in the Neotropics, *Pheidole* lineages have colonized all biogeographic realms and most of the Earth's biomes over the past approximately 37 million years. Despite the global distribution and remarkably high species richness of the genus, our analyses show that the global dominance of *Pheidole* was achieved through a series of localized radiations in different regions. There has been no exchange of lineages between the New World and Old World, little exchange between Africa, Asia and AUS–NG, and even the smaller biogeographic provinces of the Pacific (e.g. New Caledonia and Fiji) have high monophyly. This implies that *Pheidole* has either relatively low dispersal rates (just high enough for the occasional long-distance dispersal but not high enough for subsequent interchange of lineages) and/or very high incumbency; once the genus has filled niche-space in a region, new congeneric arrivals have difficulty establishing and radiating in new areas. In each region, *Pheidole* lineages have evolved *in situ* into a diversity of biomes and body sizes, exhibiting high lability in climate niche and body size evolution and a distinctive lack of phylogenetic niche conservatism on the time scales of these radiations.

The geographically clustered phylogenetic structure of *Pheidole* makes it an illuminating case study for how evolutionary radiations fill niche-space: the radiations in each geographical region form independent evolutionary experiments. In groups with a higher degree of dispersal and phylogenetic overlap among regions, it is more difficult to distinguish deterministic constraints on evolution from a shared (even if contingent) evolutionary history. Beyond the finding that *Pheidole* lineages have successfully evolved into disparate biomes in each region, the resulting pattern of variation in species richness across different biomes is strikingly similar. The re-evolution of similar patterns in different areas suggests that *Pheidole* dominance and diversity are not historical accidents, and more generally, that there are deterministic factors shaping the evolution of hyperdiversity of this group. History has repeated itself across multiple scales as *Pheidole* lineages have colonized and subsequently diversified, reaching high richness in both large (e.g. Africa, Australia) and small (e.g. Fiji, New Caledonia) geographical areas. In the Pacific archipelagoes of Fiji, Palau, Micronesia and New Caledonia, *Pheidole* is either the most species-rich or second-most species-rich genus, despite the local community being formed through various proportions of colonization and *in situ* speciation [55,56]. The fact that *Pheidole* faunas were formed through repeated radiations suggests that the high local and regional richness of *Pheidole* across the globe is due to intrinsic traits that promote diversification and ecological dominance rather than owing to random factors.

These serial *Pheidole* radiations are also structured similarly across climatic gradients within each region. Both macroecological patterns of climate–richness relationships and body size distributions have converged across the independent radiations in each East–West hemisphere. The macroecological patterns in the Old World—which resulted from a radiation of a single lineage tens of millions of years ago—are almost identical to those exhibited by *Pheidole* in the New World, with the same positive relationships between temperature and precipitation and relative magnitudes of each, and the same North–South Hemispheric asymmetry. The ability of *Pheidole* to colonize disparate geographical regions, and the lack of subsequent interchange of lineages, resulted in *Pheidole* ‘playing the tape again’ (borrowing the words of Stephen J. Gould [57]), and reaching the same endpoint, at least in terms of the macroecological structure of biodiversity. Of course, the particular climate gradient exhibited by *Pheidole*—high richness in warm, wet habitats—is not usual across taxonomic groups [16]. However, ants as a whole have been shown to peak in local richness in warm, dry, habitats [49], and individual ant genera are restricted to, or richest in, non-tropical biomes, such as *Myrmica* and *Formica* in temperate moist habitats and *Cataglyphis* and *Pogonomyrmex* in temperate dry habitats. Thus, the re-evolution of similar climate–richness patterns reflects repeatability in the way *Pheidole* genomes interact with different biomes during radiation rather than some inevitability shared by all ants.

Similarly, there are strong constraints on body size evolution that guide macroscopic patterns toward similar outcomes in different regions. Body size distributions have converged across the New World and Old World *Pheidole* despite a lack of shared lineages between the two faunas. While similar body-size distributions have evolved independently in the New and Old World *Pheidole*, it is uncertain if this conclusion of repeatability will extend to *Pheidole* phenotypes as a whole. Old World lineages have evolved aberrant spinescent phenotypes that are not found in the extant New World fauna [32], and two other Australian species were so morphologically aberrant that they were initially classified as separate monospecific genera (*Anisopheidole*, *Machomyrma*) before recently being classified as *Pheidole* owing to their phylogenetic placement within the Old World *Pheidole* radiation [41].

Our results show that evolution within and across biomes has resulted in similar climate–richness patterns in different regions, but it is as yet unknown if *Pheidole* lineages have evolved the same ecological and/or physiological adaptations allowing them to survive and thrive in different biomes. More generally, while the focus of this study was the evolution of environmental niches, there are many other dimensions of an organism's niche (e.g. diet) that were not considered and may exhibit different evolutionary patterns. While our analysis indicates that the high taxonomic richness of *Pheidole* has evolved repeatedly in both small and large biogeographic provinces, the central question of which particular adaptations are responsible for high diversification rates remains unanswered and most probably requires comparative analysis linking traits with diversification rates within and across many genera (i.e. is beyond the scope of taxa studied here).

The re-evolution of similar macroecological patterns in different regions raises interesting questions about the mechanism involved. The climate relationships cannot be explained by hard physiological limits inherited by all lineages in the genus, as individual *Pheidole* species have successfully evolved into extremely cold, dry, high latitude and high elevation habitats [21,58]. Rather, there are either top-down ecological limits on local species coexistence that vary with climate, trade-offs inherent in the *Pheidole* phenotypic schema that reduce ecological performance in cooler areas, or climate-driven differences in speciation and/or extinction rates that lead to a greater standing diversity in warm–wet areas. Further integration of ecological, phenotypic, phylogenetic and biogeographic data should allow explicit tests of these hypotheses.

## Supplementary Material

ESM

## Data accessibility

All sequences used in this study are available on GenBank, please see supplemental table for accession numbers. Collection information and images for sequenced material are available on Antweb (www.antweb.org). Richness numbers by geographical area have been uploaded to Dryad (see doi:10.5061/dryad.jk636).

## References

[1] Schipper J 2008 The status of the world's land and marine mammals: diversity, threat, and knowledge. Science 322, 225–230. (10.1126/Science.1165115) 18845749

[2] Kreft H Jetz W 2007 Global patterns and determinants of vascular plant diversity. Proc. Natl Acad. Sci. USA 104, 5925–5930. (10.1073/Pnas.0608361104) 17379667 PMC1851593

[3] Buckley LB Jetz W 2007 Environmental and historical constraints on global patterns of amphibian richness. Proc. R. Soc. B 274, 1167–1173. (10.1098//Rspb.2006.0436) PMC2189569 17327208

[4] Jetz W Rahbek C 2002 Geographic range size and determinants of avian species richness. Science 297, 1548–1551. (10.1126/Science.1072779) 12202829

[5] Orme CDL 2005 Global hotspots of species richness are not congruent with endemism or threat. Nature 436, 1016–1019. (10.1038/Nature03850) 16107848

[6] Kerkhoff AJ Moriarty PE Weiser MD 2014 The latitudinal species richness gradient in New World woody angiosperms is consistent with the tropical conservatism hypothesis. Proc. Natl Acad. Sci. USA 111, 8125–8130. (10.1073/pnas.1308932111) 24847062 PMC4050539

[7] Jetz W Thomas GH Joy JB Hartmann K Mooers AO 2012 The global diversity of birds in space and time. Nature 491, 444–448. (10.1038/nature11631) 23123857

[8] Soria-Carrasco V Castresana J 2012 Diversification rates and the latitudinal gradient of diversity in mammals. Proc. R. Soc. B 279, 4148–4155. (10.1098/rspb.2012.1393) PMC3441078 22896648

[9] Rolland J Condamine FL Jiguet F Morlon H 2014 Faster speciation and reduced extinction in the tropics contribute to the mammalian latitudinal diversity gradient. PLoS Biol. 12, e1001775 (10.1371/journal.pbio.1001775) 24492316 PMC3904837

[10] Donoghue MJ 2008 A phylogenetic perspective on the distribution of plant diversity. Proc. Natl Acad. Sci. USA 105, 11 549–11 555. (10.1073/Pnas.0801962105) 18695216 PMC2556411

[11] Cardillo M 2011 Phylogenetic structure of mammal assemblages at large geographical scales: linking phylogenetic community ecology with macroecology. Phil. Trans. R. Soc. B 366, 2545–2553. (10.1098/Rstb.2011.0021) 21807735 PMC3138611

[12] Wiens JJ 2010 Niche conservatism as an emerging principle in ecology and conservation biology. Ecol. Lett. 13, 1310–1324. (10.1111/J.1461-0248.2010.01515.X) 20649638

[13] Field R 2009 Spatial species-richness gradients across scales: a meta-analysis. J. Biogeogr. 36, 132–147. (10.1111/J.1365-2699.2008.01963.X)

[14] Mittelbach GG 2007 Evolution and the latitudinal diversity gradient: speciation, extinction and biogeography. Ecol. Lett. 10, 315–331. (10.1111/j.1461-0248.2007.01020.x) 17355570

[15] Brown JH 1995 Macroecology. Chicago, IL: University of Chicago Press.

[16] Rosenzweig M 1995 Species diversity in space and time. Cambridge, UK: Cambridge University Press.

[17] Holldobler B Wilson EO 1990 The ants. Cambridge, MA: Harvard University Press.

[18] Brady SG Schultz TR Fisher BL Ward PS 2006 Evaluating alternative hypotheses for the early evolution and diversification of ants. Proc. Natl Acad. Sci. USA 103, 18 172–18 177. (10.1073/Pnas.0605858103) PMC1838725 17079492

[19] Moreau CS 2008 Unraveling the evolutionary history of the hyperdiverse ant genus *Pheidole* (Hymenoptera : Formicidae). Mol. Phylogenet. Evol. 48, 224–239. (10.1016/J.Ympev.2008.02.020) 18394929

[20] Moreau CS Bell CD 2013 Testing the museum versus cradle tropical biological diversity hypothesis: phylogeny, diversification, and ancestral biogeographic range evolution of the ants. Evolution 67, 2240–2257. (10.1111/Evo.12105) 23888848

[21] Wilson EO 2003 Pheidole in the New World: a dominant, hyperdiverse ant genus. Cambridge, MA: Harvard University Press.

[22] Mahler DL Ingram T Revell LJ Losos JB 2013 Exceptional convergence on the macroevolutionary landscape in island lizard radiations. Science 341, 292–295. (10.1126/Science.1232392) 23869019

[23] Losos JB Jackman TR Larson A de Queiroz K Rodriguez-Schettino L 1998 Contingency and determinism in replicated adaptive radiations of island lizards. Science 279, 2115–2118. (10.1126/Science.279.5359.2115) 9516114

[24] Wagner CE Harmon LJ Seehausen O 2012 Ecological opportunity and sexual selection together predict adaptive radiation. Nature 487, 366–369. (10.1038/Nature11144) 22722840

[25] Moyle RG Filardi CE Smith CE Diamond J 2009 Explosive Pleistocene diversification and hemispheric expansion of a ‘great speciator’. Proc. Natl Acad. Sci. USA 106, 1863–1868. (10.1073/Pnas.0809861105) 19181851 PMC2644129

[26] Grant PR 1986 Ecology and evolution of Darwin‘s finches. Princeton, NJ: Princeton University Press.

[27] Schluter D 2000 The ecology of adaptive radiation. Oxford, UK: Oxford University Press.

[28] Losos JB 2009 Lizards in an evolutionary tree: the ecology of adaptive radiation in anoles. Berkeley, CA: University of California Press.

[29] Gillespie R 2004 Community assembly through adaptive radiation in Hawaiian spiders. Science 303, 356–359. (10.1126/Science.1091875) 14726588

[30] Baldwin BG Sanderson MJ 1998 Age and rate of diversification of the Hawaiian silversword alliance (Compositae). Proc. Natl Acad. Sci. USA 95, 9402–9406. (10.1073/Pnas.95.16.9402) 9689092 PMC21350

[31] Ricklefs RE 2007 History and diversity: explorations at the intersection of ecology and evolution. Am. Nat. 170, S56–S70. (10.1086/519402) 17874385

[32] Sarnat EM Moreau CS 2011 Biogeography and morphological evolution in a Pacific island ant radiation. Mol. Ecol. 20, 114–130. (10.1111/J.1365-294x.2010.04916.X) 21059129

[33] Fischer G Garcia FH Peters MK 2012 Taxonomy of the ant genus *Pheidole* Westwood (Hymenoptera: Formicidae) in the Afrotropical zoogeographic region: definition of species groups and systematic revision of the *Pheidole pulchella* group. Zootaxa 3232, 1–43.

[34] Ward PS Sumnicht TP 2012 Molecular and morphological evidence for three sympatric species of *Leptanilla* (Hymenoptera: Formicidae) on the Greek island of Rhodes. Myrmecol. News 17, 5–11.

[35] Ward PS Brady SG Fisher BL Schultz TR 2010 Phylogeny and biogeography of dolichoderine ants: effects of data partitioning and relict taxa on historical inference. Syst. Biol. 59, 342–362. (10.1093/Sysbio/Syq012) 20525640

[36] Suen G 2011 The genome sequence of the leaf-cutter ant *Atta cephalotes* reveals insights into its obligate symbiotic lifestyle. PLoS Genet. 7, e1002007 (10.1371/Journal.Pgen.1002007) 21347285 PMC3037820

[37] Nygaard S 2011 The genome of the leaf-cutting ant *Acromyrmex echinatior* suggests key adaptations to advanced social life and fungus farming. Genome Res. 21, 1339–1348. (10.1101/Gr.121392.111) 21719571 PMC3149500

[38] Rozen S Skaletsky HJ 2000 Primer3 on the WWW for general users and for biologist programmers. In Bioinformatics methods and protocols: methods in molecular biology (eds Krawetz S Misener S), pp. 365–386. Totowa, NJ: Humana Press. 10.1385/1-59259-192-2:365 10547847

[39] Drummond AJ Rambaut A 2007 BEAST: Bayesian evolutionary analysis by sampling trees. BMC Evol. Biol. 7, 214 (10.1186/1471-2148-7-214). 17996036 PMC2247476

[40] Lanfear R Calcott B Ho SY Guindon S 2012 PartitionFinder: combined selection of partitioning schemes and substitution models for phylogenetic analyses. Mol. Biol. Evol. 29, 1695–1701. (10.1093/molbev/mss020) 22319168

[41] Ward PS Brady SG Fisher BL Schultz TR 2014 The evolution of myrmicine ants: phylogeny and biogeography of a hyperdiverse ant clade (Hymenoptera: Formicidae). Syst. Entomol. (10.1111/syen.12090)

[42] Huelsenbeck JP Ronquist F 2001 MrBayes: Bayesian inference of phylogenetic trees. Bioinformatics 17, 754–755. (10.1093/Bioinformatics/17.8.754) 11524383

[43] Antweb. See http://www.antweb.org (accessed 30 December 2013).

[44] Guenard B Weiser MD Dunn RR 2012 Global models of ant diversity suggest regions where new discoveries are most likely are under disproportionate deforestation threat. Proc. Natl Acad. Sci. USA 109, 7368–7373. (10.1073/Pnas.1113867109) 22529355 PMC3358832

[45] Hijmans RJ Cameron SE Parra JL Jones PG Jarvis A 2005 Very high resolution interpolated climate surfaces for global land areas. Int. J. Climatol. 25, 1965–1978. (10.1002/Joc.1276)

[46] Gower JC Ross GJS 1969 Minimum spanning trees and single linkage cluster analysis. Appl. Stat. 18, 54–64. (10.2307/2346439)

[47] Olson DM 2001 Terrestrial ecoregions of the worlds: a new map of life on Earth. Bioscience 51, 933–938. (10.1641/0006-3568(2001)051[0933:Teotwa]2.0.Co;2)

[48] Whittaker RH 1970 Communities and ecosystems. New York, NY: Macmillan.

[49] Dunn RR 2009 Climatic drivers of hemispheric asymmetry in global patterns of ant species richness. Ecol. Lett. 12, 324–333. (10.1111/J.1461-0248.2009.01291.X) 19292793

[50] Kaspari M Ward PS Yuan M 2004 Energy gradients and the geographic distribution of local ant diversity. Oecologia 140, 407–413. (10.1007/S00442-004-1607-2) 15179582

[51] Sanders NJ Lessard JP Fitzpatrick MC Dunn RR 2007 Temperature, but not productivity or geometry, predicts elevational diversity gradients in ants across spatial grains. Glob. Ecol. Biogeogr. 16, 640–649. (10.1111/J.1466-8238.2007.00316.X)

[52] Chown SL Sinclair BJ Leinaas HP Gaston KJ 2004 Hemispheric asymmetries in biodiversity—a serious matter for ecology. PLoS Biol. 2, 1701–1707. (10.1371/journal.pbio.0020406) PMC526784 15547645

[53] Rohlf F 2013 tpsDig software v2. 17. Department of Ecology and Evolution, State University of New York at Stony Brook.

[54] Sarnat EM Rabeling C Economo EP Wilson EO In press First record of a species from the New World *Pheidole flavens*-complex (Hymenoptera: Formicidae) introduced to the southwestern Pacific. Bioinvasions Rec.

[55] Sarnat EM Economo EP 2012 The ants of Fiji. Berkeley, CA: University of California Press.

[56] Economo EP Sarnat EM 2012 Revisiting the ants of Melanesia and the taxon cycle: historical and human-mediated invasions of a tropical archipelago. Am. Nat. 180, E1–E16. (10.1086/665996) 22673659

[57] Gould SJ 1989 Wonderful life: the Burgess Shale and the nature of history. New York, NY: Norton.

[58] Bharti H Sharma YP Bharti M Pfeiffer M 2013 Ant species richness, endemicity and functional groups, along an elevational gradient in the Himalayas. Asian Myrmecol. 5, 79–101.

