# Supplementary material for: Global phylogenetic structure of the hyperdiverse ant genus Pheidole reveals the repeated evolution of macroecological patterns
Source: Proc Biol Sci. 2015 Jan 7;282(1798):20141416. doi: 10.1098/rspb.2014.1416 (PMC4262160; doi:10.1098/rspb.2014.1416)
Supplement: ESM [file rspb20141416supp1.pdf]

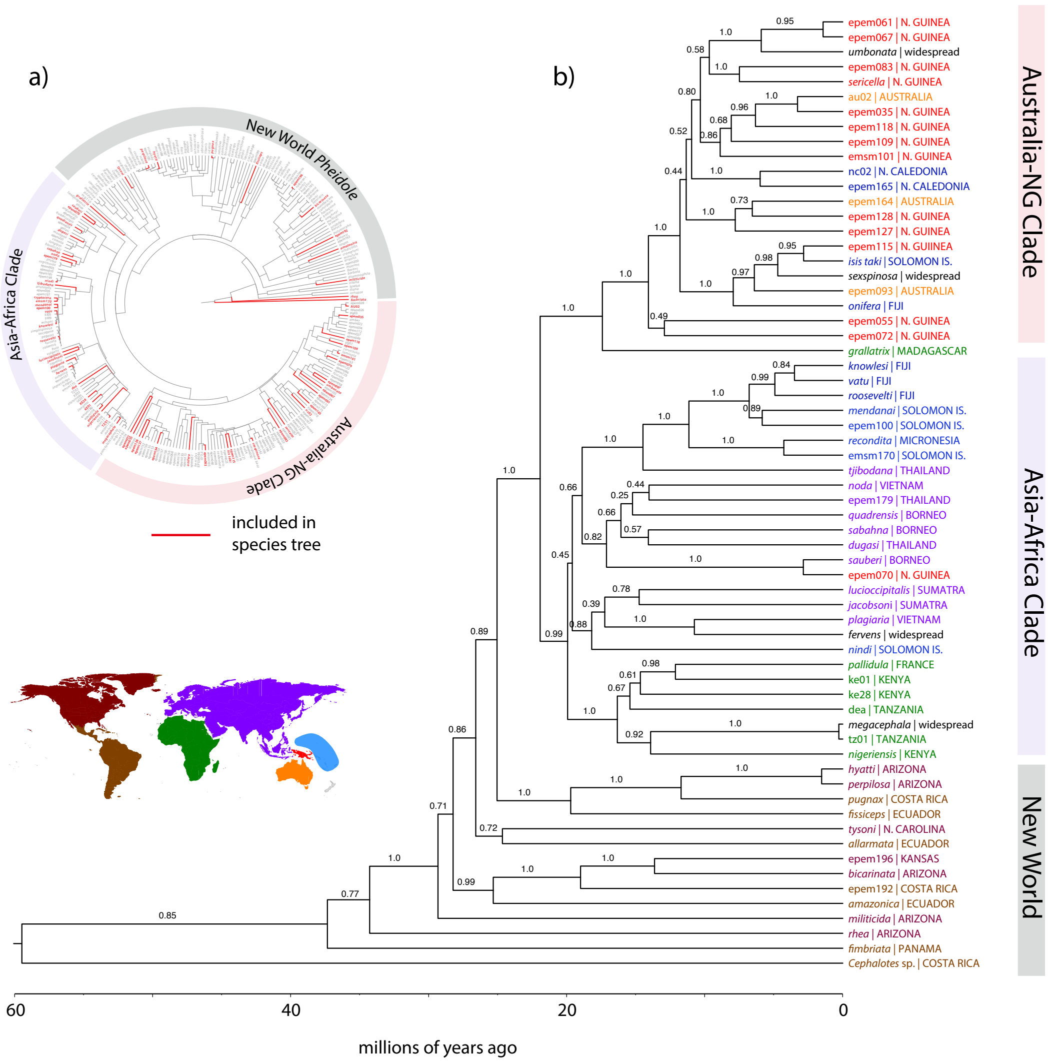

Figure S1: *Pheidole* species tree for a subset of taxa chosen to be representative of global *Pheidole* diversity. a) Selected taxa are highlighted on the tree from figure 1. b) The maximum clade credibility species tree from a posterior sample of trees generated by *BEAST based on 9 loci.

Figure S2

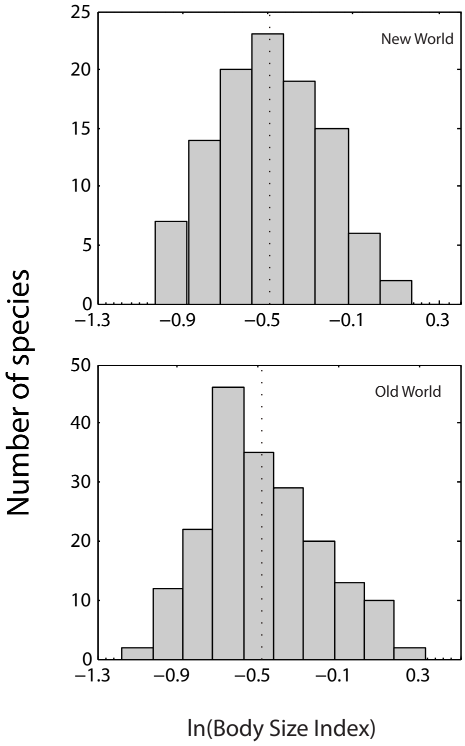

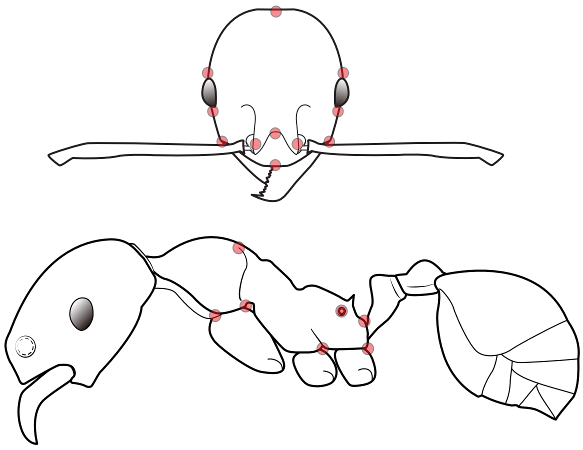

Figure S2: Body size distributions in New World and Old World regions, with the mean indicated by a vertical dashed line. The Body Size Index used here is a mean of the landmark scaling factors from a Generalized Procustes Analysis taken from the head and profile views (landmarks depicted as red dots).

Table S1: Specimens included in this study and associated GenBank accession numbers. Full collection information can be found by searching by specimen code on Antweb (www.antweb.org).

Species	Specimen Code	Collection Code	COX1	His3.3B	Lop1	GRIK2	unc-4	LOC15	CAD1	EF1a-F2	Top1
Cephalotes sp.	CASENT0283451	EGP-Z208	KJ141782	KJ141954	KJ141533	KJ141400	KJ141272	KJ141714	KJ141335	KJ141465	KJ141207
Pheidole absurda Forel	CASENT0283230	JTL3581	EF518305		EF518934						
Pheidole adrianoi Naves	CASENT0283281	ANTC24800	EF518306		EF518935						
Pheidole allarmata Wilson	CASENT0283450	ANTC22882	KJ141949	KJ142138	KJ141712	KJ141463	KJ141333	KJ141777	KJ141398	KJ141528	KJ141270
Pheidole amazonica Wilson	CASENT0283449	ANTC22881	KJ141938	KJ142127	KJ141701	KJ141459	KJ141330	KJ141773	KJ141394	KJ141524	KJ141266
Pheidole amber Donisthorpe	CASENT0219458	MJ13856	KJ141817	KJ141989	KJ141567						
Pheidole anastasii Emery	CASENT0283241	JTL3749	EF518310								
Pheidole angulicollis Eguchi	CASENT0283809	EG00-BOR-100	KJ141824	KJ141996	KJ141574						
Pheidole aristotelis Forel	CASENT0283829	MJ14128	KJ141838	KJ142010	KJ141587						
Pheidole astur Wilson	CASENT0339771	CS0410	EF518312		EF518940						
Pheidole au02	CASENT0282621	PSW15722.11	HM144334	KJ142056	KJ141632	KJ141433	KJ141305	KJ141747	KJ141368	KJ141498	KJ141240
Pheidole au04	CASENT0283190	PSW15690.18	HM144336								
Pheidole aurivillii Mayr	CASENT0283279	ANTC22845	KJ141923								
Pheidole barbata Wheeler	CASENT0283224	ANTC24795	EF518313								
Pheidole bicarinata Mayr	CASENT0283402	EMS1078	KJ141786	KJ141958	KJ141537	KJ141404	KJ141276	KJ141718	KJ141339	KJ141469	KJ141211
Pheidole biconstricta Mayr	CASENT0283253	JTL4645	EF518315		EF518942						
Pheidole bilimeki Mayr	CASENT0283244	JTL4208	EF518316								
Pheidole bluntschlii Forel	CASENT0282515	EG01-TH-666	KJ141820	KJ141992	KJ141570						
Pheidole boltoni Wilson	CASENT0283258	JTL4658	EF518318		EF518944						
Pheidole boruca Wilson	CASENT0283228	JTL3556	EF518319		EF518945						
Pheidole browni Wilson	CASENT0283247	JTL4371	EF518320		EF518946						
Pheidole bula Sarnat	CASENT0339811	EMS1791	HM144339	KJ142058	KJ141634						
Pheidole californica Mayr	CASENT0283226	ANTC24796	EF518321		EF518947						
Pheidole capellini Emery	CASENT0282516	EG01-TH-618	KJ141778	KJ141950	KJ141529						
Pheidole carrolli Naves	CASENT0339759	LRD060107-1	EF518322		EF518948						
Pheidole casta Wheeler	CASENT0339739	LRD250595	EF518323								
Pheidole cavigenis Wheeler	CASENT0283294	SPC6520	EF518324		EF518949						
Pheidole cerebrosior Wheeler	CASENT0283289	ANTC24805	EF518326		EF518951						
Pheidole ceres Wheeler	CASENT0283149	EMS1110	KJ141930	KJ142120	KJ141694						
Pheidole cervicornis Emery	CASENT0283187	MJ9389	KJ141880	KJ142050	KJ141626						
Pheidole clementensis Gregg	CASENT0339747	RAJ2303	EF518328		EF518953						
Pheidole clydei Gregg	CASENT0283293	SPC6411	EF518329		EF518954						
Pheidole cocciphaga Borgmeier	CASENT0283256	JTL4652	EF518330		EF518955						
Pheidole cockerelli Wheeler	CASENT0283295	SPC6523	EF518331		EF518956						
Pheidole colaensis Mann	CASENT0339806	EMS1784	KJ141886	KJ142059	KJ141635						
Pheidole comata Smith	CASENT0283209	Eg00-BOR-101	EF518334	KJ142074	KJ141650						
Pheidole constipata Wheeler	CASENT0339741	LRD250595	EF518335		EF518960						
Pheidole cramptoni Wheeler	CASENT0339774	CS0507	EF518336		EF518961						
Pheidole crassinoda Emery	CASENT0339736	LRD170506-6	EF518424		EF519045						
Pheidole cryptocera Emery	CASENT0283058	813.PHE.2	KJ141876	KJ142046	KJ141622	KJ141429	KJ141301	KJ141743	KJ141364	KJ141494	KJ141236
Pheidole davisi Wheeler	CASENT0339720	SPC7199	EF518337		EF518963						
Pheidole dea Santschi	CASENT0283446	ANTC22847	KJ141918	KJ142109	KJ141683	KJ141450		KJ141764	KJ141385	KJ141515	KJ141257
Pheidole dentata Mayr	CASENT0339768	CS0301	EF518338		EF518964						
Pheidole desertorum Wheeler	CASENT0339762	CS0159	EF518339		EF518965						
Pheidole diana Forel	CASENT0283246	JTL4362	EF518340		EF518966						
Pheidole distincta Donisthorpe	CASENT0199467	MJ13982	KJ141829	KJ142001	KJ141578						
Pheidole diversipilosa Wheeler	CASENT0283301	SPC7143	EF518341		EF518967						
Pheidole dossena Wilson	CASENT0283231	JTL3672	EF518342		EF518968						
Pheidole dugasi Forel	CASENT0283203	Eg01-TH-020	EF518343	KJ142075	KJ141651	KJ141440	KJ141312	KJ141754	KJ141375	KJ141505	KJ141247
Pheidole elisae Emery	CASENT0283808	EG01-TH-734	KJ141823	KJ141995	KJ141573						
Pheidole emsm101	CASENT0339807	AL0659	KJ141872	KJ142042	KJ141618	KJ141427	KJ141299	KJ141741	KJ141362	KJ141492	KJ141234
Pheidole emsm105 (nr. *striata*)	CASENT0339723	MJ1393	EF518427		EF519048						
Pheidole emsm106 (nr. *sexspinosa*)	CASENT0283509	CR111116-43	KJ141946	KJ142135	KJ141709						
Pheidole emsm107	CASENT0282492	JCM0244	KJ141797	KJ141969	KJ141548						
Pheidole emsm170	CASENT0283815	EMS2648	KJ141833	KJ142005	KJ141582	KJ141416	KJ141288	KJ141730	KJ141351	KJ141481	KJ141223
Pheidole emsm174	CASENT0339732	LRD310506-6	EF518413		EF519035						
Pheidole emsm175	CASENT0339733	LRD240506-6	EF518417		EF519039						
Pheidole emsm177	CASENT0339734	LRD140506-13	EF518421		EF519043						
Pheidole emsm180	CASENT0282712	ANTC22849	KJ141919	KJ142110	KJ141684						
Pheidole epem003	CASENT0199389	JCM0135	KJ141795	KJ141967	KJ141546						
Pheidole epem005 (nr. *rufigera*)	CASENT0199354	JCM0083	KJ141793	KJ141965	KJ141544						
Pheidole epem010	CASENT0283701	JCM0287	KJ141796	KJ141968	KJ141547						
Pheidole epem019	CASENT0283778	ANTC24776	KJ141791	KJ141963	KJ141542						
Pheidole epem020 (nr. *quadricuspis*)	CASENT0283784	ANTC22941	KJ141792	KJ141964	KJ141543						
Pheidole epem022	CASENT0199325	MJ14098	KJ141799	KJ141971	KJ141550						
Pheidole epem023 (nr. *elegans*)	CASENT0199495	MJ14107.04	KJ141806	KJ141978	KJ141556						
Pheidole epem024 (nr. *quadrensis*)	CASENT0199540	MJ14123	KJ141801	KJ141973	KJ141551						
Pheidole epem026 (nr. *bifurca*)	CASENT0199492	MJ13941	KJ141837	KJ142009	KJ141586						
Pheidole epem027 (nr. *bifurca*)	CASENT0199310	MJ14087	KJ141798	KJ141970	KJ141549						
Pheidole epem028 (nr. *bifurca*)	CASENT0282499	MJ9220	KJ141809	KJ141981	KJ141559						
Pheidole epem031	CASENT0282504	MJ14091	KJ141813	KJ141985	KJ141563						
Pheidole epem032	CASENT0199326	MJ14106	KJ141780	KJ141952	KJ141531						
Pheidole epem033	CASENT0199468	MJ13987	KJ141826	KJ141998	KJ141576						
Pheidole epem034	CASENT0199500	MJ13975	KJ141825	KJ141997	KJ141575						
Pheidole epem035 (nr. *amber*)	CASENT0199489	MJ13965	KJ141779	KJ141951	KJ141530	KJ141399	KJ141271	KJ141713	KJ141334	KJ141464	KJ141206
Pheidole epem038	CASENT0199528	MJ13776	KJ141814	KJ141986	KJ141564						
Pheidole epem040	CASENT0199512	MJ13985	KJ141827	KJ141999	KJ141577						
Pheidole epem046	CASENT0199522	MJ13983	KJ141828	KJ142000							
Pheidole epem049	CASENT0199446	MJ13950	KJ141844	KJ142016	KJ141593						
Pheidole epem050	CASENT0282503	MJ9226	KJ141812	KJ141984	KJ141562						
Pheidole epem051	CASENT0199497	MJ7493	KJ141831	KJ142003	KJ141580						
Pheidole epem055	CASENT0199533	MJ9308	KJ141816	KJ141988	KJ141566	KJ141414	KJ141286	KJ141728	KJ141349	KJ141479	KJ141221
Pheidole epem057	CASENT0339791	AL0552	KJ141865	KJ142036	KJ141613						
Pheidole epem058	CASENT0199530	MJ13973	KJ141846	KJ142018	KJ141595						
Pheidole epem060	CASENT0199477	MJ7704	KJ141845	KJ142017	KJ141594						
Pheidole epem061	CASENT0283461	MJ13801	KJ141940	KJ142129	KJ141703	KJ141461	KJ141332	KJ141775	KJ141396	KJ141526	KJ141268
Pheidole epem067 (nr. *tenuiclavata*)	CASENT0199481	MJ8576	KJ141842	KJ142014	KJ141591	KJ141418	KJ141290	KJ141732	KJ141353	KJ141483	KJ141225
Pheidole epem068	CASENT0283616	MJ9147	KJ141878	KJ142048	KJ141624						
Pheidole epem070 (nr. *elisae*)	CASENT0282484	RAPI083	KJ141783	KJ141955	KJ141534	KJ141401	KJ141273	KJ141715	KJ141336	KJ141466	KJ141208
Pheidole epem072	CASENT0339778	MJ9307	KJ141879	KJ142049	KJ141625	KJ141431	KJ141303	KJ141745	KJ141366	KJ141496	KJ141238
Pheidole epem079	CASENT0199455	MJ13935	KJ141841	KJ142013	KJ141590						
Pheidole epem083	CASENT0283824	MJ14079	KJ141835	KJ142007	KJ141584	KJ141417	KJ141289	KJ141731	KJ141352	KJ141482	KJ141224
Pheidole epem085	CASENT0199482	MJ7477	KJ141803	KJ141975	KJ141553						
Pheidole epem093	CASENT0283189	PSW15666	HM144335	KJ142057	KJ141633	KJ141434	KJ141306	KJ141748	KJ141369	KJ141499	KJ141241
Pheidole epem097	CASENT0283821	EMS2680	KJ141832	KJ142004	KJ141581						
Pheidole epem100	CASENT0283047	EMS2677	KJ141860	KJ142032	KJ141609	KJ141422	KJ141294	KJ141736	KJ141357	KJ141487	KJ141229
Pheidole epem103 (nr. *umbonata*)	CASENT0199636	EMS2661	KJ141805	KJ141977	KJ141555						

Pheidole epem109 (nr. *barumtaun*)	CASENT0283093	MJ13587	KJ141882	KJ142052	KJ141628	KJ141432	KJ141304	KJ141746	KJ141367	KJ141497	KJ141239
Pheidole epem112	CASENT0339798	RAPI087	KJ141873	KJ142043	KJ141619						
Pheidole epem113	CASENT0219468	MJ9364.1	KJ141808	KJ141980	KJ141558						
Pheidole epem115	CASENT0282485	RAPI112	KJ141785	KJ141957	KJ141536	KJ141403	KJ141275	KJ141717	KJ141338	KJ141468	KJ141210
Pheidole epem116	CASENT0199394	MJ13930	KJ141840	KJ142012	KJ141589						
Pheidole epem118	CASENT0339805	AL0657	KJ141870	KJ142040	KJ141617	KJ141426	KJ141298	KJ141740	KJ141361	KJ141491	KJ141233
Pheidole epem119	CASENT0283099	MJ9196	KJ141859	KJ142031	KJ141608						
Pheidole epem121	CASENT0283206	ANTC24798	EF518409		EF519031						
Pheidole epem123	CASENT0339793	AL0581	KJ141866	KJ142037	KJ141614						
Pheidole epem124	CASENT0283837	MJ13588	KJ141883	KJ142053	KJ141629						
Pheidole epem127 (nr. *ampla*)	CASENT0339820	MJ9266	KJ141784	KJ141956	KJ141535	KJ141402	KJ141274	KJ141716	KJ141337	KJ141467	KJ141209
Pheidole epem128 (nr. *variabilis redunca*)	CASENT0262500	MJ9364.2	KJ141810	KJ141982	KJ141560	KJ141412	KJ141284	KJ141726	KJ141347	KJ141477	KJ141219
Pheidole epem129	CASENT0339788	RAPI013	KJ141871	KJ142041							
Pheidole epem130	CASENT0283464	HP0132	KJ141884	KJ142054	KJ141630						
Pheidole epem132	CASENT0283465	HP0082	KJ141781	KJ141953	KJ141532						
Pheidole epem140	CASENT0199513	MJ14082	KJ141836	KJ142008	KJ141585						
Pheidole epem142 (nr. *sexspinosa biroi*)	CASENT0283179	HP0075	KJ141885	KJ142055	KJ141631						
Pheidole epem150	CASENT0283918	ANTC22866	KJ141914	KJ142105	KJ141681						
Pheidole epem151	CASENT0714694	ANTC22868	KJ141905	KJ142097	KJ141673						
Pheidole epem152	CASENT0283902	ANTC22865	KJ141915	KJ142106							
Pheidole epem153	CASENT0714693	ANTC22873	KJ141906	KJ142098	KJ141674						
Pheidole epem154	CASENT0283774	ANTC22873	KJ141908	KJ142100	KJ141676						
Pheidole epem155 (nr. *variabilis platypus*)	CASENT0283870	ANTC22872	KJ141911	KJ142102	KJ141678						
Pheidole epem156 (nr. *variabilis platypus*)	CASENT0283772	ANTC22873	KJ141907	KJ142099	KJ141675						
Pheidole epem157	CASENT0283843	ANTC22863	KJ141912	KJ142103	KJ141679						
Pheidole epem158	CASENT0283913	ANTC22864	KJ141916	KJ142107							
Pheidole epem159	CASENT0283770	ANTC22873	KJ141909								
Pheidole epem162	CASENT0339803	AL0637	KJ141869								
Pheidole epem163	CASENT0339801	AL0621	KJ141868	KJ142039	KJ141616						
Pheidole epem164	CASENT0283767	ANTC24810	KJ141910	KJ142101	KJ141677	KJ141449	KJ141321	KJ141763	KJ141384	KJ141514	KJ141256
Pheidole epem165 (nr. *luteipes*)	CASENT0283796	ANTC22888	KJ141849	KJ142021	KJ141598	KJ141419	KJ141291	KJ141733	KJ141354	KJ141484	KJ141226
Pheidole epem166 (nr. *variabilis aliena*)	CASENT0283791	ANTC22900	KJ141848	KJ142020	KJ141597						
Pheidole epem167	CASENT0283079	ANTC22887	KJ141858	KJ142030	KJ141607						
Pheidole epem169 (nr. *luteipes obvia*)	CASENT0283728	ANTC22903	KJ141852	KJ142024	KJ141601						
Pheidole epem170	CASENT0283797	ANTC22894	KJ141847	KJ142019	KJ141596						
Pheidole epem172	CASENT0283799	ANTC22893	KJ141851	KJ142023	KJ141600						
Pheidole epem173 (nr. *luteipes obvia*)	CASENT0282508	ANTC22875	KJ141818	KJ141990	KJ141568						
Pheidole epem174 (nr. *ampla*)	CASENT0283868	ANTC22858	KJ141942	KJ142131	KJ141705						
Pheidole epem178	CASENT0283442	EPE398	KJ141904	KJ142096	KJ141672						
Pheidole epem179 (nr. *tumida*)	CASENT0282451	EMS2772.2.01	KJ141853	KJ142025	KJ141602	KJ141420	KJ141292	KJ141734	KJ141355	KJ141485	KJ141227
Pheidole epem181	CASENT0283840	ANTC22862	KJ141913	KJ142104	KJ141680						
Pheidole epem185	CASENT0283114	EGP302	KJ141897	KJ142090	KJ141666						
Pheidole epem187	CASENT0283118	EGP301	KJ141896	KJ142089	KJ141665						
Pheidole epem188	CASENT0283481	ANTC22919	KJ141901	KJ142093	KJ141669						
Pheidole epem189	CASENT0283152	ANTC22926	KJ141900								
Pheidole epem191	CASENT0283062	MEKOU008408	KJ141934	KJ142124	KJ141698						
Pheidole epem192	CASENT0283557	EGP282	KJ141787	KJ141959	KJ141538	KJ141405	KJ141277	KJ141719	KJ141340	KJ141470	KJ141212
Pheidole epem193	CASENT0283198	EGP288	KJ141894	KJ142087	KJ141663						
Pheidole epem195	CASENT0283117	EGP290	KJ141895	KJ142088	KJ141664						
Pheidole epem196	CASENT0283804	BDB.02.N4D.M2	KJ141788	KJ141960	KJ141539	KJ141406	KJ141278	KJ141720	KJ141341	KJ141471	KJ141213
Pheidole epem197	CASENT0283517	CR111116-30	KJ141945	KJ142134	KJ141708						
Pheidole epem198 (nr. *sexspinosa*)	CASENT0283516	CR111116-19-01	KJ141943	KJ142132	KJ141706						
Pheidole epem199	CASENT0283358	CR111116-47	KJ141947	KJ142136	KJ141710						
Pheidole epem200 (cf. *moerens*)	CASENT0283317	CR111110-15	KJ141944	KJ142133	KJ141707						
Pheidole epem201	CASENT0339815	EMS2776.03.01	KJ141855	KJ142027	KJ141604						
Pheidole erratilis Wilson	CASENT0283245	JTL4334	EF518344		EF518970						
Pheidole fervens Smith	CASENT0282493	EMS1387	KJ141804	KJ141976	KJ141554	KJ141410	KJ141282	KJ141724	KJ141345	KJ141475	KJ141217
Pheidole fimbriata Roger	CASENT0283061	MEKOU010359	KJ141935	KJ142125	KJ141699	KJ141457	KJ141328	KJ141771	KJ141392	KJ141522	KJ141264
Pheidole fiorii Emery	CASENT0283264	JTL4935	EF518346		EF518971						
Pheidole fissiceps Wilson	CASENT0283448	ANTC22883	KJ141937	KJ142126	KJ141700	KJ141458	KJ141329	KJ141772	KJ141393	KJ141523	KJ141265
Pheidole fj05	CASENT0283041	EPE267.18	KJ141887	KJ142060	KJ141636						
Pheidole fj09	CASENT0283177	EPE266.14	HM144346	KJ142061	KJ141637						
Pheidole flavens Roger	CASENT0283262	JTL4928	EF518348		EF518973						
Pheidole floridana Emery	CASENT0283280	ANTC24799	EF518349		EF518974						
Pheidole furcata Sarnat	CASENT0283146	EMS2407	HM144348	KJ142062	KJ141638						
Pheidole fuscula Emery	CASENT0199529	MJ14083	KJ141802	KJ141974	KJ141552						
Pheidole gambogia Donisthorpe	CASENT0199451	MJ13940	KJ141843	KJ142015	KJ141592						
Pheidole gatesi (Wheeler)	CASENT0283204	Eg99-VN-046	EF518350	KJ142076	KJ141652						
Pheidole grallatrix Emery	CASENT0048223	BLF10792	EF518415	KJ142084	KJ141660	KJ141446	KJ141318	KJ141760	KJ141381	KJ141511	KJ141253
Pheidole harlequina Wilson	CASENT0339740	LRD250595	EF518352		EF518977						
Pheidole hercules Donisthorpe	CASENT0283830	MJ14088	KJ141839	KJ142011	KJ141588						
Pheidole hoplitica Wilson	CASENT0339728	LRD090205-10	EF518353		EF518978						
Pheidole hospes Smith	CASENT0199539	MJ13591	KJ141815	KJ141987	KJ141565						
Pheidole hyatti Emery	CASENT0283215	SPC6331	KJ141789	KJ141961	KJ141540	KJ141407	KJ141279	KJ141721	KJ141342	KJ141472	KJ141214
Pheidole impressiceps Mayr	CASENT0283220	EPE025	KJ141864								
Pheidole innupta Menozzi	CASENT0283257	JTL4656	EF518357		EF518982						
Pheidole inquilina (Wheeler)	CASENT0339753	SPC7319	EF518358		EF518983						
Pheidole isis taki Mann	CASENT0339812	EMS2593	KJ141863	KJ142035	KJ141612	KJ141425	KJ141297	KJ141739	KJ141360	KJ141490	KJ141232
Pheidole jacobsoni Forel	CASENT0283213	SMP4974	EF518410	KJ142082	KJ141658	KJ141445	KJ141317	KJ141759	KJ141380	KJ141510	KJ141252
Pheidole jelskii Mayr	CASENT0283274	SPCDR-33	EF518359		EF518984						
Pheidole juniperae Wilson	CASENT0339727	LRD160805-16	EF518360		EF518985						
Pheidole ke01	CASENT0283278	ANTC22849	KJ141924	KJ142114	KJ141688	KJ141452	KJ141323	KJ141766	KJ141387	KJ141517	KJ141259
Pheidole ke03	CASENT0283318	ANTC22845	KJ141925	KJ142115	KJ141689						
Pheidole ke08	CASENT0283275	ANTC22852	KJ141926	KJ142116	KJ141690						
Pheidole ke28	CASENT0283064	ANTC22853	KJ141928	KJ142118	KJ141692	KJ141453	KJ141324	KJ141767	KJ141388	KJ141518	KJ141260
Pheidole knowlesi Mann	CASENT0283124	EMS2055	HM144350	KJ142063	KJ141639	KJ141435	KJ141307	KJ141749	KJ141370	KJ141500	KJ141242
Pheidole laminata Emery	CASENT0283823	MJ14089	KJ141834	KJ142006	KJ141583						
Pheidole laselva Wilson	CASENT0283268	JTL5221	EF518361		EF518986						
Pheidole laticornis Wilson	CASENT0283229	JTL3566	EF518362								
Pheidole liengmei Forel	CASENT0283068	ANTC22848	KJ141920	KJ142111	KJ141685						
Pheidole littoralis Cole	CASENT0339760	LRD140107-4	EF518363		EF518987						
Pheidole longipes (Latreille)	CASENT0283807	EG98-BOR-815	KJ141822	KJ141994	KJ141572						
Pheidole lucioccipitalis Eguchi	CASENT0283208	SMP5073	EF518418	KJ142085	KJ141661	KJ141447	KJ141319	KJ141761	KJ141382	KJ141512	KJ141254
Pheidole macrops Wilson	CASENT0339749	RAJ3012	EF518364		EF518988						
Pheidole macula Wheeler	CASENT0283299	SPC7091	EF518368		EF518991						
Pheidole megacephala (Fabricius)	CASENT0283042	802.PHE.1	KJ141874	KJ142044	KJ141620	KJ141428	KJ141300	KJ141742	KJ141363	KJ141493	KJ141235
Pheidole sericella Viehmeyer	CASENT0282497	MJ14090	KJ141807	KJ141979	KJ141557	KJ141411	KJ141283	KJ141725	KJ141346	KJ141476	KJ141218
Pheidole mendanai Mann	CASENT0283049	EMS2678	KJ141861	KJ142033	KJ141610	KJ141423	KJ141295	KJ141737	KJ141358	KJ141488	KJ141230
Pheidole metallescens Emery	CASENT0339724	ANTC24806	EF518367		EF518990						
Pheidole mg110	CASENT0077599	BLF10145	EF518422		EF519044						
Pheidole mgs123	CASENT0498508	BLF10051	EF518425		EF519046						
Pheidole mg162	CASENT0283200	CS0242	KJ141892	KJ142083	KJ141659						
Pheidole militicida Wheeler	CASENT0283148	EMS1159	KJ141932	KJ142122	KJ141696	KJ141456	KJ141327	KJ141770	KJ141391	KJ141521	KJ141263

Pheidole minutula Mayr	CASENT0283227	ANTC24797	EF518370		EF518993						
Pheidole moerens Wheeler	CASENT0283221	LRD290304-9	EF518371		EF518994						
Pheidole montana Eguchi	CASENT0282518	EG98-BOR-847	KJ141821	KJ141993	KJ141571						
Pheidole morrisii Forel	CASENT0283306	SPC7198	EF518372		EF518995						
Pheidole multispina Wilson	CASENT0283529	K16.CAT600RAH	KJ141917	KJ142108	KJ141682						
Pheidole nc02	CASENT0283182	ANTC22874	KJ141888	KJ142064	KJ141640	KJ141436	KJ141308	KJ141750	KJ141371	KJ141501	KJ141243
Pheidole nigeriensis Santschi	CASENT0283555	ANTC22849	KJ141929	KJ142119	KJ141693	KJ141454	KJ141325	KJ141768	KJ141389	KJ141519	KJ141261
Pheidole nindi Mann	CASENT0283055	EPE197	KJ141862	KJ142034	KJ141611	KJ141424	KJ141296	KJ141738	KJ141359	KJ141489	KJ141231
Pheidole nitella Wilson	CASENT0283261	JTL4925	EF518373		EF518996						
Pheidole nitidicollis Emery	CASENT0283265	JTL5201	EF518374		EF518997						
Pheidole noda Smith	CASENT0283210	Eg-99-VN-039	EF518375	KJ142077	KJ141653	KJ141441	KJ141313	KJ141755	KJ141376	KJ141506	KJ141248
Pheidole obscurithorax Naves	CASENT0283225	LRD050304-4	EF518376		EF518999						
Pheidole obtusospinosa Pergande	CASENT0339757	SPC7413	EF518377		EF519000						
Pheidole oceanica Mayr	CASENT0283054	811.PHE.1	KJ141875	KJ142045	KJ141621						
Pheidole onifera Man	CASENT0283163	EMS2472	KJ141889	KJ142065	KJ141641	KJ141437	KJ141309	KJ141751	KJ141372	KJ141502	KJ141244
Pheidole pallidula (Nylander)	CASENT0283201	F(31) Lauragais-2001	EF518381	KJ142078	KJ141654	KJ141442	KJ141314	KJ141756	KJ141377	KJ141507	KJ141249
Pheidole pegasus Sarnat	CASENT0283142	EMS2370	HM144355	KJ142066	KJ141642						
Pheidole pelor Wilson	CASENT0339725	LRD250606-21	EF518382		EF519005						
Pheidole perpilosa Wilson	CASENT0283508	EMS1058	KJ141931	KJ142121	KJ141695	KJ141455	KJ141326	KJ141769	KJ141390	KJ141520	KJ141262
Pheidole pg01	CASENT0283165	EMS680	HM144356								
Pheidole pg02	CASENT0283167	EMS729	HM144357	KJ142067	KJ141643						
Pheidole pg03	CASENT0283168	EMS793	HM144358	KJ142068	KJ141644						
Pheidole pilifera (Roger)	CASENT0283270	PSW15165	EF518380		EF519003						
Pheidole plagiaria Smith	CASENT0283212	Eg99-VN-008	KJ141891	KJ142079	KJ141655	KJ141443	KJ141315	KJ141757	KJ141378	KJ141508	KJ141250
Pheidole polymorpha Wilson	CASENT0283266	JTL5206	EF518389		EF519012						
Pheidole portalensis Wilson	CASENT0339748	RAJ2613	EF518388		EF519011						
Pheidole protea Forel	CASENT0283303	Eg01-TH-084	EF518390								
Pheidole psammophila Creighton & Gregg	CASENT0339744	RAJ1157	EF518391		EF519013						
Pheidole psilogaster Wilson	CASENT0283238	ANTC22929	KJ141903	KJ142095	KJ141671						
Pheidole pubiventris Mayr	CASENT0283243	JTL4054	EF518356		EF518981						
Pheidole pugnax Dalla Torre	CASENT0283558	EGP278	KJ141893	KJ142086	KJ141662	KJ141448	KJ141320	KJ141762	KJ141383	KJ141513	KJ141255
Pheidole punctulata spinosa Forel	CASENT0076779	BLF10229	EF518419		EF519041						
Pheidole purpurea Longino	CASENT0283153	ANTC22918	KJ141899	KJ142092	KJ141668						
Pheidole quadrensis Forel	CASENT0283205	Eg00-BOR-129	EF518392	KJ142080	KJ141656	KJ141444	KJ141316	KJ141758	KJ141379	KJ141509	KJ141251
Pheidole quadricuspis Emery	CASENT0283211	Eg-00-BOR-037	EF518393	KJ142081	KJ141657						
Pheidole quadrispinosa (Smith)	CASENT0339795	AL0613	KJ141867	KJ142038	KJ141615						
Pheidole radoszkowskii Mayr	CASENT0283147	JCM0518	KJ141936								
Pheidole rhea Wheeler	CASENT0283452	ANTC22886	KJ141939	KJ142128	KJ141702	KJ141460	KJ141331	KJ141774	KJ141395	KJ141525	KJ141267
Pheidole rhinoceros Forel	CASENT0283263	JTL4932	EF518396		EF519018						
Pheidole roosevelti Mann	CASENT0283120	EMS1968	HM144366	KJ142069	KJ141645	KJ141438	KJ141310	KJ141752	KJ141373	KJ141503	KJ141245
Pheidole rufescens Wheeler	CASENT0339754	SPC4783	EF518397		EF519019						
Pheidole rugithorax Eguchi	CASENT0283827	EMS2772.2.02	KJ141854	KJ142026	KJ141603						
Pheidole rugulosa Gregg	CASENT0283251	JTL4503	EF518398		EF519020						
Pheidole sabahna Eguchi	CASENT0282514	EG98-BOR-850	KJ141819	KJ141991	KJ141569	KJ141415	KJ141287	KJ141729	KJ141350	KJ141480	KJ141222
Pheidole sauberi Forel	CASENT0282502	MJ14126	KJ141811	KJ141983	KJ141561	KJ141413	KJ141285	KJ141727	KJ141348	KJ141478	KJ141220
Pheidole sciara Cole	CASENT0339751	RAJ3185	EF518399		EF519021						
Pheidole sciophila Wheeler	CASENT0283271	PSW15141	EF518400		EF519022						
Pheidole scrobifera Emery	CASENT0283249	JTL4407	EF518401		EF519023						
Pheidole sebofila Longino	CASENT0283252	JTL4643	EF518355		EF518980						
Pheidole senex Gregg	CASENT0283296	SPC6591	EF518402		EF519024						
Pheidole sensitive Borgmeier	CASENT0283254	JTL4647	EF518403		EF519025						
Pheidole sericella Viehmeyer	CASENT0282497	MJ14090	KJ141807	KJ141979	KJ141557	KJ141411	KJ141283	KJ141725	KJ141346	KJ141476	KJ141218
Pheidole sexdentata Donisthorpe	CASENT0283802	MJ8239	KJ141857	KJ142029	KJ141606						
Pheidole sexspinosa biroi Emery	CASENT0199306	MJ6268	KJ141800	KJ141972							
Pheidole sexspinosa Mayr	CASENT0199371	JCM0156	KJ141794	KJ141966	KJ141545	KJ141409	KJ141281	KJ141723	KJ141344	KJ141474	KJ141216
Pheidole sicaria Wilson	CASENT0283248	JTL4391	EF518405		EF519027						
Pheidole simonsi Wilson	CASENT0283070	ANTC22928	KJ141902	KJ142094	KJ141670						
Pheidole simplispinosa Sarnat	CASENT0283131	EMS2097	KJ141890	KJ142070	KJ141646						
Pheidole singularis Smith	CASENT0283059	MJ13580	KJ141881	KJ142051	KJ141627						
Pheidole sitiens Wilson	CASENT0283284	LRD080205-13	EF518406		EF519028						
Pheidole soritis Wheeler	CASENT0283298	SPC7085	EF518407		EF519029						
Pheidole sospes Forel	CASENT0339824	ANTC24793	EF518408		EF519030						
Pheidole spadonia Wheeler	CASENT0283288	ANTC24804	EF518428		EF519049						
Pheidole tandjongensis Forel	MCZ-ENT00511508	EG01-TH-064	EF518431		EF519051						
Pheidole tepicana Pergande	CASENT0283292	SPC6348	EF518432		EF519052						
Pheidole texticeps Wilson	CASENT0283526	ANTC22923	KJ141898	KJ142091	KJ141667						
Pheidole titanis Wheeler	CASENT0339726	LRD030805-4	EF518434		EF519054						
Pheidole tjibodana Forel	CASENT0283800	EMS2776.03.04	KJ141856	KJ142028	KJ141605	KJ141421	KJ141293	KJ141735	KJ141356	KJ141486	KJ141228
Pheidole tristicula Wilson	CASENT0339770	CS0402	EF518435		EF519055						
Pheidole tysoni Forel	CASENT0283454	ANTC22884	KJ141790	KJ141962	KJ141541	KJ141408	KJ141280	KJ141722	KJ141343	KJ141473	KJ141215
Pheidole tz01	CASENT0283066	ANTC22854	KJ141921	KJ142112	KJ141686	KJ141451	KJ141322	KJ141765	KJ141386	KJ141516	KJ141258
Pheidole tz02	CASENT0282918	ANTC22855	KJ141927	KJ142117	KJ141691						
Pheidole ug01	CASENT0283158	ANTC22856	KJ141922	KJ142113	KJ141687						
Pheidole umbonata Mayr	CASENT0283086	827.PHE.1	KJ141877	KJ142047	KJ141623	KJ141430	KJ141302	KJ141744	KJ141365	KJ141495	KJ141237
Pheidole umbonata Mayr	CASENT0199400	JCM0217H	KJ141948	KJ142137	KJ141711	KJ141462		KJ141776	KJ141397	KJ141527	KJ141269
Pheidole umphreyi Wilson	CASENT0283259	JTL4838	EF518439		EF519059						
Pheidole uncagena Sarnat	CASENT0283143	EMS2372	HM144377	KJ142071	KJ141647						
Pheidole vallicola Wheeler	CASENT0283063	EMS1062	KJ141933	KJ142123	KJ141697						
Pheidole vatu Mann	CASENT0283101	EMS1841.9	HM144378	KJ142072	KJ141648	KJ141439	KJ141311	KJ141753	KJ141374	KJ141504	KJ141246
Pheidole violacea Wilson	CASENT0283255	JTL4649	EF518442		EF519063						
Pheidole vistana Forel	CASENT0283272	PSW15142	EF518443		EF519064						
Pheidole vorax (Fabricius)	CASENT0339773	CS0506	EF518325		EF518950						
Pheidole vulgaris Eguchi	CASENT0283455	EG01-VN-234	KJ141941	KJ142130	KJ141704						
Pheidole wilsoni Mann	CASENT0283162	EMS2459.1	HM144383	KJ142073	KJ141649						
Pheidole xanthocnemis Emery	CASENT0283725	ANTC22890	KJ141850	KJ142022	KJ141599						
Pheidole xanthogaster Wilson	CASENT0339769	CS0399	EF518444		EF519065						
Pheidole xerophila Wheeler	CASENT0339764	CS0224	EF518437		EF519057						
Pheidole yaqui Creighton & Gregg	CASENT0339750	RAJ3083	EF518446		EF519067						

Table S2. PCR protocols and oligonucleotide primer sequences to amplify 9 loci of different *Pheidole* species

**Locus** [a]	**Len**[b]	**Primer Name** [c]	**Sequence** (5'->3')	**Anneal**	**Extension** [d]
CO1Lc.parent	1.20	PHE_TyrBF	TTTACAATTTATTACTTTWATCAGACA	48	72
		PHE_CO1_1163R	GAAATCAATGRATRAATCTRGCAAT	0:35	2:15+1s/cycle
CO1Lc.child	1.20	PHE_TyrCF_T	TGTAAAACGACGGCCAGTTTTATTACTTTWATCAGACATAAYACT	50	72
		PHE_CO1_1141BR_T	CAGGAAACAGCTATGACCATAGCRAATACAGCHCCYAT	0:30	2:00
H3Ls.parent	0.60	PHE_H3_863F	BSGTVMRTCCCCACCACT	49	72
		PHE_H3_1438R	AAWTTTTGGCCCTGAAAAGG	0:35	1:00
H3Ls.child	0.58	PHE_H3_866F_T	TGTAAAACGACGGCCAGTGTVMRTCCCCACCACTAGC	52	72
		PHE_H3_1429R_T	CAGGAAACAGCTATGACCCCTGAAAAGGGCCGATTG	0:35	0:50
Lop1.parent	1.13	PHE_Lop1_1081F	CAAACRGTGGTSGACAAAGTG	49	72
		PHE_Lop1_3793R	GAGARCCCCARATYGTGAAGAG	0:35	2:00
Lop1.child	1.11	PHE_Lop1_1093F_T	TGTAAAACGACGGCCAGTAAAGTGCCACCGGAGATG	51	72
		PHE_Lop1_3785R_T	CAGGAAACAGCTATGACCRATYGTGAAGAGIGGGCTGA	0:30	1:40
CG3822.parent	0.90	PHE_CG3822_2839F	GAAGCAGATCTSGCBATCAC	49	72
		PHE_CG3822_3967R	AARAACCACCARCARCTTGC	0:35	2:15+1s/cycle
CG3822.child	0.90	PHE_CG3822_2845_T	TGTAAAACGACGGCCAGTATCTSGCBATCACGGATCT	51	72
		PHE_CG3822_3967R_T	CAGGAAACAGCTATGACCCACCARCARCTTGCMAYCAT	0:35	1:50
OdsH2.parent	1.05	PHE_OdsH_4218F	CGAGAGACARCAGCGGAAG	49	72
		PHE_OdsH_6051R	TGGTGRTTCARRTGCTCCAT	0:35	2:15+1s/cycle
OdsH2.child	0.95	PHE_OdsH_4236F_T	TGTAAAACGACGGCCAGTAGCTCGCTGCTAAAGTAAGC	51	72
		PHE_OdsH_5993R_T	CAGGAAACAGCTATGACCGAGCGCYGGGAAACATTC	0:35	1:50
GEN15.parent	1.13	PHE_GEN15_1024F	ACCCATCGGTTTTGCATT	49	72
		PHE_GEN15_2314R	ATCTTYCRACTCGTCATCCA	0:35	2:15+1s/cycle
GEN15.child	1.10	PHE_GEN15_1033F_T	TGTAAAACGACGGCCAGTTCGGTTTTGCATTTAGTGATTG	51	72
		PHE_GEN15_2306R_T	CAGGAAACAGCTATGACCACTCGTCATCCATTGCATTC	0:35	1:50
CAD1.parent	0.99	PHE_CAD1_274F	CTYAGCAAYGGWCCRGGTGA	49	72
		PHE_CAD1_1952R	TCCGGTRTAATYGGYAAGAAA	0:35	2:15+1s/cycle
CAD1.child	0.99	PHE_CAD1_286F_T	TGTAAAACGACGGCCAGTCCRGGTGATCCYARCATGTG	51	72
		PHE_CAD1_1932R_T	CAGGAAACAGCTATGACCGAAATAYACTTTRTCRGCCATKCC	0:35	1:50
EF1a_F2.parent	1.60	PHE_EF1a1-F2_218F	AATGYGGTGGTATYGACAAGC	49	72
		PHE_EF1a1-F2_1875R	AACRACCATACCRGGTTTYA	0:35	2:15+1s/cycle
EF1a_F2.child	1.60	PHE_EF1a1-F2_255F_T	TGTAAAACGACGGCCAGTGAGAARGAAGCCCAGGAGGT	51	72
		PHE_EF1a1-F2_1864R_T	CAGGAAACAGCTATGACCCCRGGTTTYAGTACACCRGTC	0:35	1:50
TOP1.parent	0.94	PHE_TOP1_1655F	TKCAGGTGGGARGARGARAAGAA	52	72
		PHE_TOP1_2593R	GTYACCTAARAARTCRAABACRAC	0:35	2:15+1s/cycle
TOP1.child	0.84	PHE_TOP1_1709F_T	TGTAAAACGACGGCCAGTGARCAYAARGGACCKGTRTTYGCACC	51	72
		PHE_TOP1_2512R_T	CAGGAAACAGCTATGACCGARCARCARCCYACDGTRTCHGCYTG	0:35	1:10
